# Home on the Range: Factors Explaining Partial Migration of African Buffalo in a Tropical Environment

**DOI:** 10.1371/journal.pone.0036527

**Published:** 2012-05-03

**Authors:** Robin Naidoo, Pierre Du Preez, Greg Stuart-Hill, Mark Jago, Martin Wegmann

**Affiliations:** 1 Conservation Science Program, World Wildlife Fund-United States (WWF-US), Washington, D.C. United States of America; 2 Directorate of Scientific Services, Ministry of Environment and Tourism, Windhoek, Namibia; 3 World Wildlife Fund (WWF) in Namibia, Windhoek, Namibia; 4 Department of Remote Sensing, University of Wuerzburg, Wuerzburg, Bavaria, Germany; University of Alberta, Canada

## Abstract

Partial migration (when only some individuals in a population undertake seasonal migrations) is common in many species and geographical contexts. Despite the development of modern statistical methods for analyzing partial migration, there have been no studies on what influences partial migration in tropical environments. We present research on factors affecting partial migration in African buffalo (*Syncerus caffer*) in northeastern Namibia. Our dataset is derived from 32 satellite tracking collars, spans 4 years and contains over 35,000 locations. We used remotely sensed data to quantify various factors that buffalo experience in the dry season when making decisions on whether and how far to migrate, including potential man-made and natural barriers, as well as spatial and temporal heterogeneity in environmental conditions. Using an information-theoretic, non-linear regression approach, our analyses showed that buffalo in this area can be divided into 4 migratory classes: migrants, non-migrants, dispersers, and a new class that we call “expanders”. Multimodel inference from least-squares regressions of wet season movements showed that environmental conditions (rainfall, fires, woodland cover, vegetation biomass), distance to the nearest barrier (river, fence, cultivated area) and social factors (age, size of herd at capture) were all important in explaining variation in migratory behaviour. The relative contributions of these variables to partial migration have not previously been assessed for ungulates in the tropics. Understanding the factors driving migratory decisions of wildlife will lead to better-informed conservation and land-use decisions in this area.

## Introduction

Partial migration occurs when a fraction of an animal population migrates to and from disjunct seasonal home range areas, while the remainder of individuals remain on one home range the entire year [1]. Hypotheses for partially migratory behaviour may be divided into two broad (and potentially overlapping) classes. Individuals within a population may be genetically predisposed to one or the other strategy [2]. Alternatively, animals within a population may show conditional migratory behaviour that depends on variation in individual (age, sex, behaviour), social (conspecific densities, position in dominance hierarchy), or environmental (predation risk, availability of resources such as food and breeding sites) factors [3]. Partial migration has been documented in a variety of taxa, including mammals [3], [4], [5], fish [6], birds [7], and amphibians [8].

The development of cheaper, more durable and more accurate satellite tracking technology has advanced our knowledge of animal movements in a variety of ways [9], [10]. In addition to hardware advances, new statistical approaches for modeling the large quantities of georeferenced location data emanating from tracking devices continue to be proposed [11], [12], [13], including methods to explicitly quantify and classify migratory behaviour within a sample of tagged animals [14]. This combination of enhanced technology and greater analytical capabilities has proven instrumental in advancing the field of movement ecology, and similarly has begun to deepen our understanding of partial migration [3], [4], [5].

Ungulates are an ecologically and economically important group of animals that have received a relatively large share of research attention in terms of modern animal movement studies [15], [16]. Nevertheless, our current understanding of partial migration in ungulates is limited in two important ways. Firstly, research has been largely conducted in temperate areas [16], [17], [18]. Yet understanding partial migration in tropical ungulates is important for a variety of reasons, perhaps primarily because the migration of large ungulates drives ecosystem dynamics in a number of emblematic and globally significant conservation landscapes [19], [20]. In addition, while insights from temperate studies can and should be used to infer results in tropical settings, fundamental differences between the two regions necessitate conducting primary studies in the tropics. In particular, seasonality in temperate zones is defined largely by differences in temperature, whereas seasonality in the tropics (especially in savannas) is defined primarily by differences in precipitation [21]. As a result, ungulates' access to drinking water is a more strongly limiting factor in tropical environments than in most temperate ones [22], [23], and may therefore shape migratory behaviours in fundamentally different ways than in temperate climates.

Unlike studies on space use in ungulates [24], [25], most investigations of partial migration have to-date been largely descriptive and have not taken advantage of modern statistical modeling approaches to quantify factors affecting migratory behaviour. There has been relatively little consideration of how environmental or other factors influence the propensity of individuals to migrate, and/or the distance to which they do so, despite recognition of the importance of this topic [26]. Without such information, conservation or management strategies will likely be insufficiently informed to account for the effects of changing drivers such as climate and land use on partially migratory species.

Here, we address many of these issues by studying how partial migration in African buffalo (*Syncerus caffer*), a large ungulate of economic and ecological significance in tropical African savannas [27], [28], varies according to a range of putative drivers. We first use recent statistical methods to classify individuals into several migratory classes, and also propose a new class in addition to those mentioned in Bunnefeld *et al.* 2010 [14]. Secondly, we use multiple regression models to quantify the relative impact of a variety of environmental conditions and individual-specific characteristics on metrics of migratory status. Our results highlight the importance of environmental heterogeneity in conditioning partial migration in ungulate populations, a result which has to-date received little attention in the literature.

## Methods

### Study area

**Figure 1:**
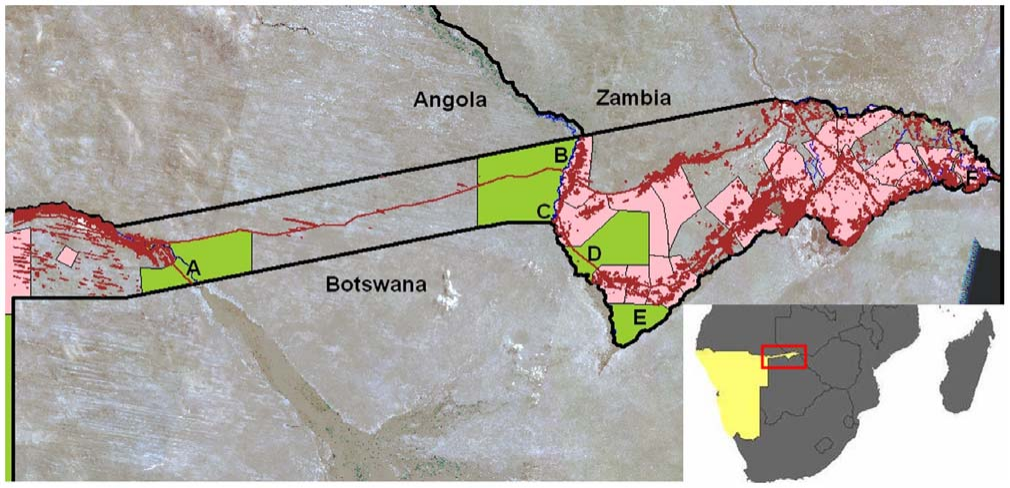
The study area and buffalo GPS locations. The study area was the Caprivi Strip of Namibia, with GPS locations of *n* = 31 collared animals (different colours indicate different individuals). In addition, green indicates protected areas, pink areas are communal conservancies, and brown lines indicate major roads. Capture sites are (A) Mahango National Park/Buffalo Core Conservation Area; (B) Susuwe; (C) Horseshoe; (D) Mudumu National Park; (E) Mamili National Park; (F) Eastern Floodplains.

The study area was the Caprivi Strip, a thin (∼30 km at its narrowest point) strip of land running east-west in the northeast corner of Namibia, as well as surrounding areas of Angola and Botswana (Fig. 1). Topography in the Caprivi is flat (930–1100 metres above sea level), and rainfall averages around 650 mm per year, mostly falling between November and April. The average daily temperature is approximately 23° Celsius.

The Caprivi Strip is at the centre of the proposed Kavango-Zambezi Transfrontier Conservation area (KAZA), an enormous agglomeration of existing and proposed protected areas that will take in parts of five countries, and aims to capitalize on the region's outstanding nature-based tourism potential to drive livelihood gains in a highly impoverished part of Africa. Human populations in the Caprivi are very high compared to most of the rest of Namibia: 115,000 people as of 1996 [29]. Tensions between conservation and development, in the form of anthropogenic barriers to wildlife movements, as well as human-wildlife conflict around crops and livestock, pose a challenge to implementing the ambitious vision of KAZA as a conservation-based driver of human livelihood gains. In this context, assessing migratory behaviours and resulting connectivity in the region is of critical importance.

Within this region we focused on quantifying partial migration and environmental conditions at six study sites, from west to east: Mahango National Park/Buffalo Core Conservation Area, Susuwe, Horseshoe, Mudumu National Park, Mamili National Park, and the Eastern Floodplains (Fig. 1). Mamili National Park and the Eastern Floodplains are predominantly grassland habitats, whereas the other sites are a mix of floodplain grasslands, treed savannas, and some woodlands. While topography in the study area varies little (from 930 to 1100 metres above sea level), there is strong seasonality in terms of precipitation (most of the ∼650 mm of rain falls during a wet season from November to April), and the sites also exhibit temporal and spatial heterogeneity of other environmental characteristics, as well as in potential boundaries to movement.

### Field methods

Field work was conducted at the end of the dry season (late September – mid October) in three years: 2007, 2009, and 2010. Adult buffalo (*n* = 31, of which 26 were females) were darted from a helicopter and immobilized using a mixture of etorphine hydrochloride, azaperone, and hyaluronidase. Age of each individual and the size of the herd that the animal was embedded in at capture were estimated. All 32 collars (one individual was recaptured after two years and had its non-functioning collar replaced) were programmed to record Global Positioning System (GPS) locations at 5-hour intervals. Animals were captured and treated according to the protocols approved under research permits 1184/2007, 1339/2008, and 1537/2010 from the Ministry of Environment and Tourism, Namibia.

### Seasonality and partial migration

Many species in nearby areas, including elephants [30] and buffalo [31] show seasonal space use patterns. In the dry season, animals range up and down floodplains and adjacent woodlands several kilometers from permanent rivers, whereas they move away from rivers and into areas containing ephemeral water sources in distant woodlands during the wet season. To quantify the prevalence of this behaviour in buffalo of the Caprivi Strip, we calculated the distance to the nearest river for all GPS locations, and used recently developed statistical methods [14] to classify individuals according to which of four competing models of migratory behaviour best explained temporal patterns in distance to river. The four competing models were:

#### Non-migratory



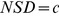
(1)


#### Nomadic



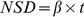
(2)


#### Disperser



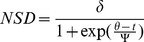
(3)


#### Migratory



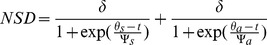
(4)


As described in [14], the parameters in the above equations represent the following:

*c*, *β* – Constants

*t* – Time (days)

*δ* – Distance (metres) at which NSD asymptotes

*θ* – Time at which migration reaches half the asymptotic distance (subscripted *s* for first move away from dry season range, and *a* for move back to dry season range)

*ψ* – Time elapsed between reaching half and three-quarters of migration (subscripted *s* for first move away from dry season range, and *a* for move back to dry season range)

Since models described by equations (3) and (4) are non-linear in the time predictor variable, we used non-linear regression methods, implemented in the nls library of the statistical package R [32] to produce Akaike Information Criterion (AIC) scores for each model. For each individual buffalo, the lowest AIC score identified which of the four competing models had the greatest support given the data. We classified each individual into one of the four classes according to this best-fitting model. Furthermore, we used Akaike weights [33] to quantify the probability or likelihood that this model was the best.

The parameter *θ* was used as the breakpoint to divide chronosequences of migrating and dispersing individuals into dry seasons and wet seasons. For individuals that did not display any distinct seasonality to their movements, we subdivided chronosequences of GPS observations using the average seasonal breakpoints of migratory individuals, so that all 32 individual GPS chronosequences were subdivided into distinct seasons. For example, if wet season movements in 2007 for 3 migratory individuals were determined to begin, using the methods described above, on November 5, November 13, and November 15, non-migratory individuals were assumed to be in the “wet season” as of November 11. This design resulted in the identification of 47 discrete dry seasons that were followed by wet season movements. We characterized several measures of wet season migratory behaviour: average and maximum distance away from permanent rivers in the wet season, average and maximum Net Squared Displacement from point of capture (which always occurred in the dry season), with higher values of each assumed to reflect a greater degree of migratory behaviour.

### Variables affecting migration

Our interest was in characterizing environmental conditions in the dry season and then using these measures to explain variation in migratory/non-migratory behaviour in the subsequent wet season. We therefore represented dry season space use by calculating Local Convex Hull (LoCoH) home ranges. Simulation analyses have shown that LoCoH produces more robust and biologically meaningful estimates of space utilization in animals as compared to Minimum Convex Polygons and kernel approaches [34], [35]. In particular, LoCoH is superior to kernel methods in dealing with boundaries or regions that are biologically inaccessible to individual animals [35]. As with other home range methods, various isopleth levels can be calculated with LoCoH; we used only the 90% isopleth in our models, a level that has been recommended for home range studies [35], [36].

We then characterized environmental conditions within LoCoH dry season home ranges using a set of variables that, based on the literature and our own knowledge of the study area, we believed might play a role in conditioning partial migration. Most of these were variables related to the external environment, but we also included several to control for differences among individual buffalo, their social environment, physical constraints on movement, and differences in sample size of GPS readings (Table 1).

**Table 1:** Descriptive statistics from *n* = 32 GPS chronosequences from African buffalo in the Caprivi Strip of Namibia.

Sex	Region	Home range size	Net Squared Displ. (km) 2		Distance nearest river (km)		Best model	Akaike weight	Sample size
		(km^2^)^1^	Mean	Max	Mean	Max			
Female	buffalo	73.45	6.24	16.58	3.18	15.27	expander	1	1332
Female	susuwe	322.21	18.84	37.35	7.86	28.29	migrant	1	2132
Female	susuwe	221.75	20.25	37.07	10.65	26.28	migrant	0.99	303
Female	buffalo	104.67	6.03	12.05	3.12	13.00	expander	1	2240
Female	susuwe	260.85	16.33	40.35	6.97	29.53	migrant	1	1289
Female	mamili	155.72	8.26	16.47	5.76	11.77	non-migrant	0.87	1797
Female	mudumu	242.95	15.28	40.41	8.41	36.93	expander	1	2799
Female	mamili	84.07	7.29	17.91	6.90	12.48	non-migrant	0.48	3328
Female	susuwe	318.71	17.79	37.22	11.55	28.38	migrant	1	1202
Male	susuwe	125.47	10.66	29.18	4.79	20.85	migrant	1	241
Male	eflood	6.48	4.54	6.44	1.03	3.11	-3		69
Male	eflood	3.95	3.59	17.93	0.44	16.95	disperser	1	756
Male	horseshoe	3.95	7.57	12.74	2.34	3.72	-3		63
Female	horseshoe	448.34	75.64	106.75	56.80	81.23	disperser	1	1235
Female	eflood	5.46	3.13	6.59	0.45	2.84	non-migrant	1	1038
Female	eflood	15.54	3.50	6.84	0.58	2.79	disperser	0.88	2223
Female	mudumu	241.06	21.52	34.31	16.02	37.57	disperser	0.88	2098
Female	mudumu	153.04	16.16	30.37	20.13	37.09	non-migrant	1	702
Male	mamili	0.60	3.13	5.16	3.85	6.78	-3		22
Female	mamili	76.89	8.32	17.54	7.07	12.62	non-migrant	0.87	3431
Female	buffalo	74.25	6.86	22.36	2.71	12.48	migrant	1	2138
Female	horseshoe	564.74	78.12	114.90	46.91	81.74	migrant	1	1195
Female	mudumu	192.04	20.80	41.09	17.52	38.67	expander	1	1314
Female	mudumu	200.97	14.49	34.07	11.68	36.71	non-migrant	0.94	1109
Female	buffalo	242.14	27.03	44.31	27.86	45.96	migrant	1	1197
Female	susuwe	287.99	13.62	28.42	24.61	37.59	migrant	1	1154
Female	mamili	62.94	7.10	18.12	9.79	17.44	non-migrant	1	1212
Female	buffalo	103.06	8.40	14.72	4.98	12.83	expander	1	1148
Female	mamili	50.75	7.96	16.49	10.40	17.58	non-migrant	0.82	813
Female	susuwe	280.51	23.87	40.04	15.71	38.34	migrant	1	1060
Female	susuwe	229.63	24.54	45.31	24.01	39.01	migrant	1	1280
Female	horseshoe	422.58	52.43	74.53	44.78	74.83	migrant	1	1213

1 LoCoNH 90% isopleth, calculated for all points across all seasons in each chronosequence.

2 Note that we take the square root of the raw Net Squared Displacement so that distances are more easily interpreted.

3 Insufficient observations to classify.

Vegetation affects animal movements because of differences in resource availability, susceptibility to predation, and ease of travel [24], [37], [38], [39]. We constructed three separate variables that represented vegetation structure and habitat quality. First, we used a new dataset (M. Wegmann *et al.*, unpublished data) derived from MODIS data that estimates the percent tree cover in each 250-m cell on the landscape. We summarized (mean and standard deviation) the percent tree cover for each dry season home range. Secondly, we used the 250-m resolution MODIS Enhanced Vegetation Index (EVI), a measure of the greenness of the vegetation in each cell and a correlate of vegetation biomass [38], and calculated the mean and standard deviation for each dry season home range. Finally, we used a map [29] that classified vegetation structure in the Caprivi Strip region into nine categories (wetland, high closed woodland, high open woodland, tall open grassland, tall closed woodland, tall closed grassland, tall open woodland, high closed shrubland, and high closed grassland). We calculated the proportion of dry season home range covered by each of these vegetation types, and from this extracted the dominant vegetation type in each home range.

Abiotic environmental conditions may affect movement rates of large herbivores in various ways. Rainfall may increase movements by supplying water to formerly dry areas, but may decrease space use by increasing heat loss in individuals [15], [24], [40]. Similarly, fires may cause animals to range further in searching out flushes of new grass that follow burns (when sufficient moisture is present to allow vegetation regrowth), but without such regrowth fires may have a negative effect on habitat selection [40], [41]. We used remotely sensed data from the Tropical Rainfall Monitoring Mission (TRMM) to characterize precipitation experienced by each collared buffalo. Daily precipitation data were acquired (http://trmm.gsfc.nasa.gov/) at a resolution of 0.25°×0.25° and the rainfall intensity (mm of rain/day) was summarized for each dry season home range. MODIS daily fire locations (product “MOD14”, at a resolution of 1 km×by 1 km) were acquired from https://wist.echo.nasa.gov, and the frequency of fire events was summarized for each home range.

Natural (rivers) and anthropogenic (fences, human settlements, agricultural areas) potential barriers to animal movement are found throughout the study area. We calculated a proxy for the level of “constraint” that a buffalo may experience when faced with a decision to migrate or not by using three potential barriers to movement. For each GPS location of each individual we calculated the distance to the nearest permanent river, the distance to the nearest fence, and the distance to the nearest agricultural clearing. We took the minimum of these three distances for each GPS location, and then averaged these minima for each dry season home range. We assume that smaller values of this distance-barrier-variable reflect greater possible constraints on migratory behaviour.

The age of an animal has variously been positively, negatively, or not at all related to space use of large herbivores [24]. We therefore included age as a predictor in our models of migration. In addition, herd size has in some instances been correlated with home range size in buffalo, and may reflect the immediate social environment that is important to a species with a strong social structure [28], [42]. We included the estimated size of the herd that the collared individual was in at the time of capture as an additional possible predictor of migration.

Finally, to control for possible effects of sample size, we included as a covariate the number of GPS readings in the wet season, assuming that animals with more GPS readings were, all else equal, likely to have greater distances moved.

### Statistical analysis

The migration metrics of average and maximum distance to river, and average and maximum NSD, were all highly correlated with one another (r>0.9, *p*<<0.001). We therefore chose maximum NSD as a continuous dependent variable representing the degree to which an individual migrated, with greater NSD's indicative of longer seasonal migrations. We log-transformed this variable to reduce deviations from normality in its distribution. To aid in interpreting the eventual regression coefficients, we scaled each predictor variable by subtracting the mean and dividing by two standard deviations [43]. Prior to formal statistical analyses, correlation analysis, scatterplots, and boxplots were used to assess potential collinearity among scaled predictor variables. There was a strong correlation between the average percent tree cover in a home range and the dominant vegetation type in a home range. We therefore removed the latter categorical variable and retained the continuous tree cover variable for analysis. No other severe collinearity among variables was detected. However, the distribution of the number of fires per home range was extremely skewed, with 32 of 47 dry season home range estimates having no fires, and the remaining 15 home ranges having from 1 to 111 fires. Because the extreme imbalance in this variable makes its predictive power low, we transformed it into a dichotomous variable, assigning the value “1” if any number of fires occurred in a home range, and “0” if not.

To quantify the relative effect of each dry season variable on subsequent wet season migration distance, we used an information-theoretic approach [33] to assess the 4096 candidate models that included all possible combinations of predictor variables described above (including a fire∶rainfall interaction term). For each model we used ordinary least squares multiple regression and calculated Akaike weights based on the small sample size AIC (AICc); these were used to calculate importance values and model-averaged coefficients for each variable. All analyses described in the paper were conducted using the statistical software R [32], in particular using the packages adehabitat (Nearest Neighbor Convex Hull script) for home range estimations [44] and AICcmodavg for multi-model inference.

## Results

**Figure 2:**
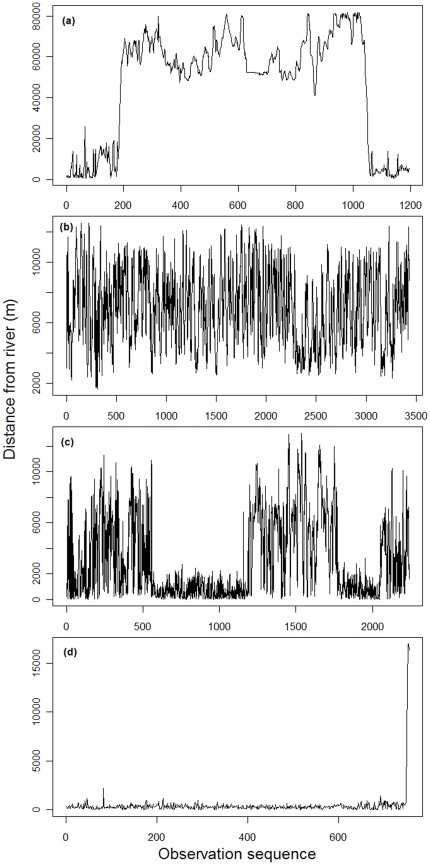
Examples of seasonal movement types displayed by adult buffalo in the Caprivi region of Namibia. (a) migrant; (b) non-migrant; (c) expander; (d) disperser.

Individual buffalo displayed variable migratory behaviour, which we classified into four broad types. Following Bunnefeld *et al.* [14], migrants were identified as those individuals showing a disjunct seasonal space use pattern, with dry season ranges alongside permanent watercourses and geographically disjunct wet season ranges up to 100 km distant in woodlands and savannas (Table 1, Fig. 2a). In contrast, non-migrants showed no such seasonal pattern in distance from rivers (Fig. 2b). Post-hoc examination of individuals initially classified as migratory revealed that a subset displayed episodic wet season forays away from permanent watercourses and into savanna/woodlands, but not to the same distance, length of time, or degree of geographical separation from their dry season range as in true migrants (Fig. 2c). The average coefficient of variation for distance to rivers in the wet season was significantly larger in this class as compared to migrants (mean expanders = 0.82, mean of migrants = 0.37, *F* = 19.4, *p* = 0.0003), and minimum distances to rivers in the wet season were substantially smaller (mean expanders = 81.5 metres, mean migrants = 9745 metres, Tukey HSD, *F* = 3.6, *p* = 0.07). We label this intermediate class, not discussed in Bunnefeld *et al.* [14], as “expanders” since they expanded (rather than moved entirely away from) their dry season home ranges during the wet season (*n* = 6). Four individuals were classified as dispersers, including one male buffalo that crossed the Chobe river and ventured directly south into Botswana before the collar stopped transmitting (Fig. 2d). For three individuals, the duration of time that the collar functioned was not long enough to permit characterization. For the 11 individuals whose data record spanned more than 12 months (i.e., individuals that were tracked across multiple wet and/or dry seasons), the same migratory strategy was always displayed, i.e., individuals either always migrated or always did not migrate, but never switched from one strategy to the other. Akaike weights for competing models showed that there was typically strong evidence for one “best” movement class model, rather than similar support for many of the competing models (Table 1 column “Akaike weight”; mean Akaike weight = 0.95, standard deviation = 0.11).

For the three classes with sufficient data to permit comparisons over all observations, migrants had the largest home ranges and had the greatest average and maximum distances away from rivers and away from collaring location (Table 2). Non-migrants had the smallest of all of these measures, with expanders intermediate in scale but not significantly different from either migrants or non-migrants, except that their home range size was smaller than that of migrants. During the dry season, migrants again had larger home ranges than non-migrants (with expanders intermediate and not significantly different), though removal of the individuals in the Eastern Floodplains (who all had extremely small home ranges) resulted in no significant dry season range differences among classes.

**Table 2:** Differences in movement metrics among migratory behaviour classes for African buffalo in the Caprivi Strip of Namibia. Differences in superscripts indicate statistically significant differences (Tukey's post-hoc HSD test).

Variable	Individual class		
	Migratory	Expander	Non-migratory
Home range (km^2^)^1^	292.24^a^	159.5^b^	89.5^b^
Mean net squared displacement (km)^2^	29.69^a^	13.0^ab^	8.46^b^
Maximum net squared displacement (km)	50.6^a^	26.5^ab^	18.2^b^
Average distance to nearest river (km)	21.93^a^	8.87^ab^	8.08^b^
Maximum distance to nearest river (km)	41.89^a^	25.7^ab^	16.8^b^
Dry season home range (km^2^)^1^	110.6^a^	78.2^ab^	52.3^b^

1 LoCoH 90% isopleth.

2 Note that we take the square root of the raw Net Squared Displacement so that distances are more easily interpreted.

The information-theoretic approach identified only a small number of important models among the 4096 tested. The top 10 models accounted for 48% of the cumulative Akaike weight (Table 3), while the top 30 models (0.7% of the total number of models) accounted for over 75% of the cumulative Akaike weight. These results, along with the consistency of variables contained in the best models of Table 3, are indicative of a consensus set of important predictor variables.

**Table 3:** Characteristics of ten best models explaining buffalo migration (dependent variable = Net Squared Displacement) in the Caprivi Strip of Namibia.

Model no.	Variables in Candidate Model^1^	AICc	Delta AICc	Akaike Weight	Cumulative Weight	Log Likelihood
3325	EVI.wtd.sd, frac_mean, frac_sd, Dist.barrier.avg, rain.mm.day, fires, herd, EVI	55.89	0	0.09	0.09	−14.89
3827	EVI.wtd.sd, frac_mean, frac_sd, Dist.barrier.avg, rain.mm.day, fires, herd, EVI, EVIsq	56.23	0.34	0.07	0.16	−13.35
2995	frac_mean, frac_sd, Dist.barrier.avg, rain.mm.day, fires, herd, EVI	56.73	0.84	0.06	0.22	−16.93
2659	EVI.wtd.sd, frac_mean, Dist.barrier.avg, rain.mm.day, fires, herd, EVI	56.9	1.01	0.05	0.27	−17.02
4027	EVI.wtd.sd, frac_mean, frac_sd, Dist.barrier.avg, rain.mm.day, log(ss), fires, herd, EVI, EVIsq	57.22	1.33	0.05	0.32	−12.02
3805	EVI.wtd.sd, frac_mean, frac_sd, Dist.barrier.avg, rain.mm.day, log(ss), fires, herd, EVI	57.58	1.69	0.04	0.36	−14.02
3458	EVI.wtd.sd, frac_mean, Dist.barrier.avg, rain.mm.day, fires, herd, EVI	57.72	1.83	0.04	0.39	−15.8
3455	EVI.wtd.sd, frac_mean, Dist.barrier.avg, rain.mm.day, fires, age, rain.fire.int, herd	57.79	1.9	0.03	0.42	−15.84
3662	frac_mean, frac_sd, Dist.barrier.avg, rain.mm.day, fires, herd, EVI, EVIsq	57.95	2.06	0.03	0.46	−15.92
3823	EVI.wtd.sd, frac_mean, Dist.barrier.avg, rain.mm.day, log(ss), fires, herd, EVI	58.2	2.31	0.03	0.48	−16.04

1 frac_mean = Proportion of home range in woodlands; rain.mm.day = Average rainfall on dry season home range (mm); Dist.barrier.avg = Distance to nearest linear barrier (river, fence, or cultivated area), metres; EVIsq = Square of EVI variable; age = Animal's age at capture (years); fires = Binary variable indicating presence of fires on dry season home range; log(ss) = Number of wet season GPS observations (log-transformed); herd = Size of animal's herd at capture; rain∶fire int = Interaction variable of rainfall and fire presence; EVI.wtd.sd = Standard deviation of EVI variable; EVI = Average EVI value on dry season home range; frac_sd = Standard deviation of frac_mean variable.

**Figure 3:**
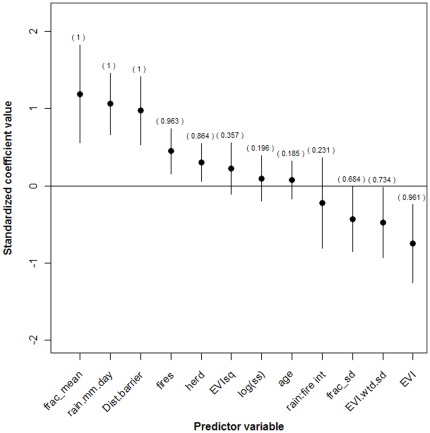
Results of statistical modeling. Model-averaged (over the set of 4096 candidate regression models) standardized regression coefficients for variables explaining wet season migratory movements in African buffalo. Variable abbreviations: frac_mean = Proportion of home range in woodlands; rain.mm.day = Average rainfall on dry season home range (mm); Dist.barrier.avg = Distance to nearest linear barrier (river, fence, or cultivated area), metres; EVIsq = Square of EVI variable; age = Animal's age at capture (years); fires = Binary variable indicating presence of fires on dry season home range; log(ss) = Number of wet season GPS observations (log-transformed); herd = Size of animal's herd at capture; rain∶fire int = Interaction variable of rainfall and fire presence; EVI.wtd.sd = Standard deviation of EVI variable; EVI = Average EVI value on dry season home range; frac_sd = Standard deviation of frac_mean variable.

A plot of the standardized variable coefficients shows the identity of the most important predictors (Fig. 3). Dry season home range variables that had a positive effect on subsequent migration distance were the percent tree cover, daily rainfall, distance from barriers, presence of fires, and size (at capture) of the animal's herd. In contrast, EVI and variability in both EVI and percent tree cover in a dry season home range had negative effects on subsequent migration distance.

We repeated the above analyses for the subset of home ranges that corresponded to individuals in only the migrant, expander, and disperser classes (i.e., removing non-migrants from the analysis). The direction on all variable coefficients remained the same, and the relative importance of variables was very similar (Fig. S1). As opposed to the full analysis, the only differences were that the positive coefficients on the incidence of fires and the size of the herd now had 95% confidence intervals that overlapped with zero.

## Discussion

Individual buffalo in our study area displayed three of the migratory patterns that were described in Bunnefeld *et al.* [14]: migratory, non-migratory, and dispersing. However, we also observed a fourth class of pattern not considered in the framework of Bunnefeld *et al.* [14]: “expander” individuals whose wet season home ranges continued to incorporate their dry season range, but expanded to also include woodland areas away from permanent watercourses. The extent to which this is a pattern specific to buffalo in this study area or a general pattern among tropical ungulates is unclear, and will not be resolved without more case studies of the type we present here. Nevertheless, we argue that the five movement types used to characterize migratory behaviour in ungulates in Bunnefeld *et al.* [14] are incomplete and that the expander category we have documented warrants further consideration and investigation as a class of migratory movement that may be present in other study systems.

**Figure 4:**
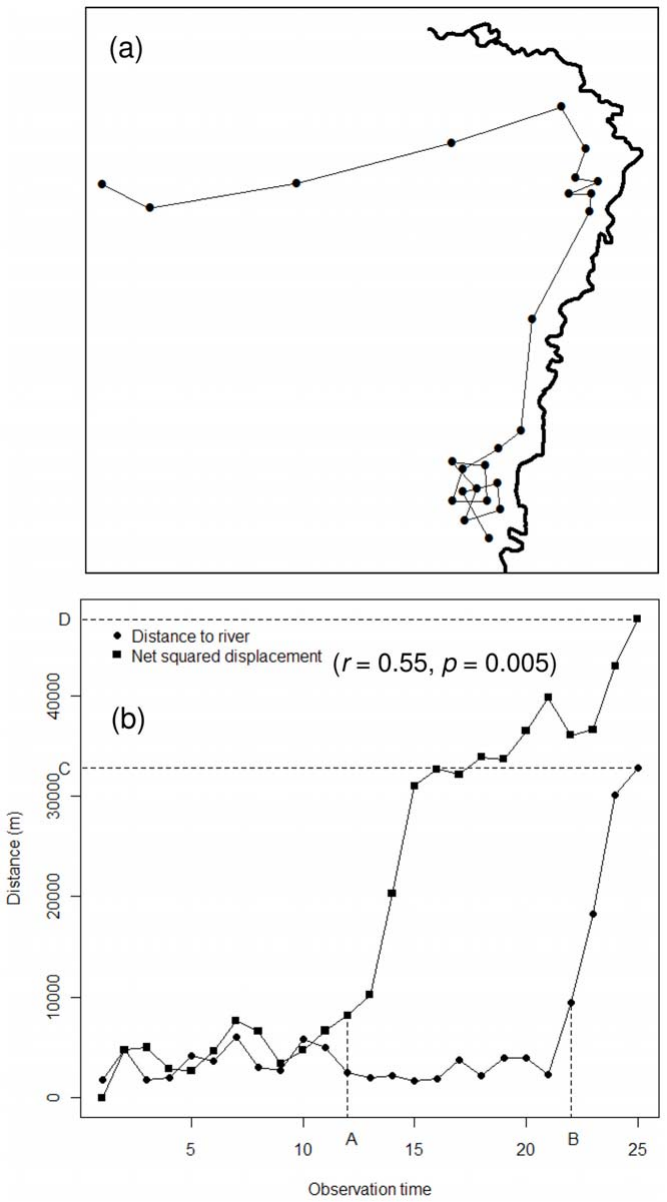
Inference of migratory behaviour from net-squared displacement. (a) Schematic of an animal moving alongside a permanent river (heavy line) during the dry season, and then migrating to a wet season range; and (b) the resulting plots of distance to river vs observation time and net-squared displacement (NSD) vs. observation time (A – onset of migration inferred from NSD; B – onset of migration inferred from distance to river; C – distance migrated inferred from distance to river; D – distance migrated inferred from NSD).

Although modern statistical methods documenting and explaining partial migration have been used for a variety of species and ecosystems, examples from the tropics are almost entirely with reference to birds [7], [45], and examples from tropical ungulates are non-existent as far as we are aware. Our work highlights several ways in which studies of partial migration in the tropics may differ in significant ways from those in temperate areas. In the first instance, the use of NSD as a standalone measure of migratory behaviour may lead to potentially erroneous inferences regarding timing of migration in tropical ungulates that display seasonal movements alongside and then away from rivers. A simple example shows how the linearity of dry season movements alongside rivers seen in our case study, and common among many surface water-constrained tropical ungulates [46], could result in an underestimate of the timing of seasonal migrations using NSD, even when both metrics are strongly correlated, as they were in our data (Fig. 4). In these instances quantifying the timing of migration by using the distance to the river along which animals were captured (assuming dry season captures) is likely to result in more accurate inferences regarding migratory behaviour than will using NSD. On the other hand, NSD remains a consistent metric that can be compared among individuals captured at different initial distances away from rivers, and therefore remains an appropriate metric to measure the length of subsequent migratory movements.

Secondly, in addition to permanent watercourses, ephemeral water sources are important resources for tropical herbivores in strongly seasonal environments such as savannas [22], [23], [30]. Unfortunately, while some mapping of water holes in the study region has been conducted [47], we do not have a comprehensive GIS layer of all water points that are available to buffalo throughout our study area. Anecdotally, omurambas (depressions that fill with water in the wet season) are more abundant and better developed in the Susuwe area compared to the Buffalo/Mahango and Horseshoe areas. They are not present at all at the other study sites, though there are a number of artificial boreholes, along with numerous, largely unmapped, water points, in Mudumu National Park. Interpreting and quantifying all of these water sources in a spatially consistent manner would allow a direct estimation of how the availability of ephemeral water sources compares to the availability of forage as determinants of migratory behaviour. Unfortunately, current remote sensing technology makes detection of these water points at the necessary spatial and temporal scales prohibitively expensive, as it would require numerous fine-scale (e.g., 5-m resolution Quickbird) satellite images at various time periods for the entire study area. We estimate it would have cost close to $9 million USD to purchase these satellite data.

Despite its possible importance [28], [40], [42], we were also unable to quantitatively evaluate the relative effects of predation risk on the propensity of buffalo in our study to migrate. Data on the distribution and/or density of lions (*Panthera leo*), the principal predator of buffalo in the study area, is not available except in anecdotal form from a preliminary survey conducted in 2011 (O. Aschenborn, Ministry of Environment and Tourism, Namibia, personal communication). Under a strong effect of lion predation on migratory behaviour, we would expect that non-migratory individuals in Mamili National Park and the Eastern Floodplains would be experiencing the lowest predation risk, whereas highly mobile individuals in Horseshoe, Susuwe, and to some degree the Buffalo Core Area, would be under the highest predation risk. However, lion densities in the Caprivi are apparently, from highest to lowest: Mamili, Mudumu, Horseshoe, Susuwe, Mahango, Buffalo, Eastern Floodplains. When available, future work should include quantitative measures of lion densities into models such as we have described here to examine more rigorously how predation risk compares to other environmental conditions in driving migratory behaviour in buffalo.

In addition to the points about the tropics that we raise above, there has been a general focus in the partial migration literature on description and characterization rather than on prediction or drivers. Our results are consistent with the hypothesis that the availability and quality of forage exerts a dominant effect on the degree to which individuals move away from dry season ranges. Specifically, a greater availability of grasslands (the inverse of the percent tree cover in a home range) and a lower quality of forage (as proxied for by EVI) leads individuals to move further away from their dry season ranges. In addition, the more variable EVI and grasslands are in dry season home range, the less far buffalo migrate in the wet season. As with other African grazers, the availability of forage is a key resource for buffalo that has been demonstrated to affect movement and space use in various geographical contexts [28], [40], [41].

Important abiotic environmental predictors of wet season migration distance were dry season home range estimates of the amount of rainfall and the presence of fires. Both of these variables had positive effects on migration distance. Buffalo and other ungulates of seasonal savannas are thought to be able to track rainfall events over large distances [48], [49] Higher rainfall experienced on dry season home ranges may thus spur animals to migrate further away from rivers due to a higher expected probability of encountering ephemeral drinking water sources, upon which they depend while away from permanent rivers. In contrast, the presence of fires may be acting independently to “push” buffalo off unfavorable dry season ranges. Buffalo in Tanzania's Serengeti National Park generally avoided both grasslands and woodlands that had been burned, only returning to these areas weeks or months after fires [40]. Similarly, in Zimbabwe buffalo were not associated with recently burned areas, unlike most of the other ungulate species examined [50]. Our results provide no support for the “greenflush” hypothesis, i.e., an attractor effect of the flush of new grass that follows rainfall on new burns [40], [41], which would have been indicated by a negative coefficient on the rain∶fire interaction term in our models. However, this hypothesis is probably better tested at the level of the individual movement step by modeling parameters such as step length and turning angle in relation to proximity to nearest burn, while controlling for the other environmental variables we have included here.

Our analysis also showed that non-environmental factors affect buffalo migration. As expected, individuals that were less constrained by barriers on their dry season home ranges migrated further than those nearer to barriers. At the level of the individual 5-hour step our data also show (informally) that fences and rivers block buffalo movements, while certain parts of the main tar road bisecting the Caprivi Strip also act as a barrier. As with elephants in this region [51], buffalo are inherently mobile herbivores that are nevertheless constrained by linear barriers such as rivers, fences, and roads, as well as by the presence of human settlements and cultivation. Although the buffalo movements in our study region were some of the longest on record for non-dispersing individuals [52], this was at least partly a function of anthropogenic barriers shaping movement trajectories. For example, the fence marking the southern border between Botswana and Namibia, along with the tar road and associated human settlement, acted to funnel a buffalo collared in the Horseshoe area over 100 km west down a narrow ∼15 km channel, with an eventual retracing of its steps and ultimately a descent into Botswana through a 20 km gap in the border fence (Video S1). These barriers rendered potential habitat to the south in Botswana and north of the tar road in Namibia inaccessible during the wet season, and therefore may have increased the distance ultimately traveled by this buffalo, with the consequent increases in energetic expenditures and predator risk.

In addition, animals in larger herds at the time of capture migrated further than those in smaller herds. Buffalo in Kruger National Park also ranged more widely when in larger herds [28]. If this is indeed a general phenomenon, this has implications for further development in the KAZA transfrontier conservation area. The continued development of agricultural areas and settlements, especially along rivers, are increasingly denying habitat to buffalo, with attendant negative consequences for population size [53]. Smaller population numbers and smaller herd sizes are likely to mean smaller migratory movements, which will independently reduce connectivity and gene flow among increasingly separated sub-populations.

KAZA aims to boost livelihoods in this region by increasing nature-based tourism. Since a key driver of tourism in this region is wildlife, ensuring the persistence of migratory and/or highly mobile species is important not only for conservation, but also from a human development point of view. We have shown here that both environmental conditions and anthropogenic barriers affect buffalo movements in this partially migratory population. Despite the stated aims of KAZA existing barriers such as game fences and an increasing human population with its attendant infrastructure and land use change pose a major challenge to wildlife connectivity. Assuming, as elsewhere [54], [55], that other wide-ranging and (partially) migratory species may be experiencing reduced connectivity due to these anthropogenic factors, careful attention to connectivity conservation will be necessary in order for sustainable development to proceed in the KAZA region.

## Supporting Information

Video S1 **Long-range migratory movements of two female buffalo.** An animation of a year's worth of movements from two female buffalo collared in the Horseshoe area of the Caprivi Strip, Namibia.
(WMV)

Figure S1 **Results of statistical modeling when excluding non-migratory individuals.** Model-averaged (over the set of 4096 candidate regression models) standardized regression coefficients for variables explaining wet season migratory movements in African buffalo. Variable abbreviations: frac_mean = Proportion of home range in woodlands; rain.mm.day = Average rainfall on dry season home range (mm); Dist.barrier.avg = Distance to nearest linear barrier (river, fence, or cultivated area), metres; EVIsq = Square of EVI variable; age = Animal's age at capture (years); fires = Binary variable indicating presence of fires on dry season home range; log(ss) = Number of wet season GPS observations (log-transformed); herd = Size of animal's herd at capture; rain∶fire int = Interaction variable of rainfall and fire presence; EVI.wtd.sd = Standard deviation of EVI variable; EVI = Average EVI value on dry season home range; frac_sd = Standard deviation of frac_mean variable.
(TIF)
