# Supplementary figures and images for: Home on the Range: Factors Explaining Partial Migration of African Buffalo in a Tropical Environment
Source: PLoS One. 2012 May 3;7(5):e36527. doi: 10.1371/journal.pone.0036527 (PMC3343005; doi:10.1371/journal.pone.0036527)

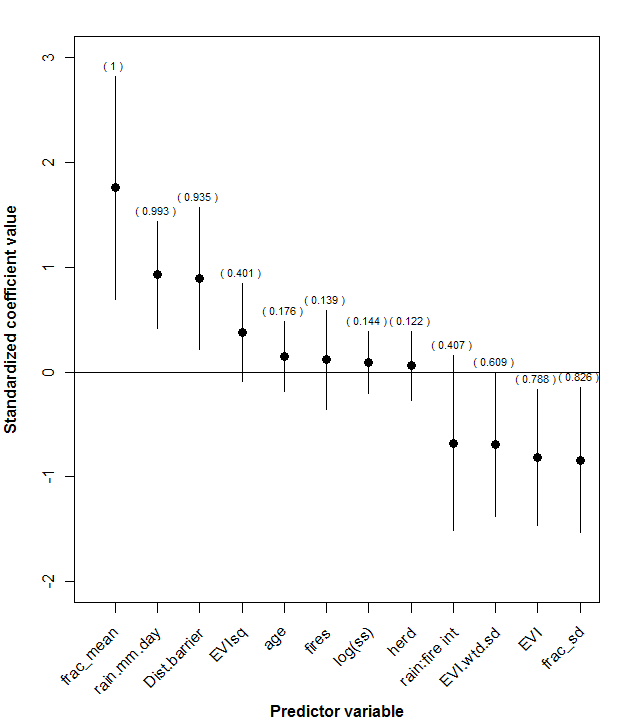

Supplement: Figure S1 — Results of statistical modeling when excluding non-migratory individuals. Model-averaged (over the set of 4096 candidate regression models) standardized regression coefficients for variables explaining wet season migratory movements in African buffalo. Variable abbreviations: frac_mean = Proportion of home range in woodlands; rain.mm.day = Average rainfall on dry season home range (mm); Dist.barrier.avg = Distance to nearest linear barrier (river, fence, or cultivated area), metres; EVIsq = Square of EVI variable; age = Animal's age at capture (years); fires = Binary variable indicating presence of fires on dry season home range; log(ss) = Number of wet season GPS observations (log-transformed); herd = Size of animal's herd at capture; rain∶fire int = Interaction variable of rainfall and fire presence; EVI.wtd.sd = Standard deviation of EVI variable; EVI = Average EVI value on dry season home range; frac_sd = Standard deviation of frac_mean variable. (TIF) [file pone.0036527.s002.tif]
